# Whole-genome sequencing reveals a previously unrecognized measles virus cluster in Burundi

**DOI:** 10.1371/journal.pone.0351691

**Published:** 2026-06-30

**Authors:** Néhémie Nzoyikorera, David F. Nieuwenhuijse, Leonard Schuele, Cassien Nduwimana, Armstrong Ndihokubwayo, Théogène Ihorimbere, Denis Niyomwungere, Hayley Cassidy, Marjan Boter, Celestin Nibogora, Richard Molenkamp, Alexis Niyomwungere, Dionis Nizigiyimana, Marie Noelle Uwineza, Idrissa Diawara, Saria Otani, Rogier Bodewes, Frank M. Aarestrup, Marion Koopmans, Joseph Nyandwi, Bas B. Oude Munnink

**Affiliations:** 1 National Reference Laboratory, National Public Health Institute, Bujumbura, Burundi; 2 Department of Viroscience, Erasmus University Medical Center, Rotterdam, the Netherlands; 3 Laboratory of Clinical Immunology, Infection and Autoimmunity (LICIA), Faculty of Medicine and Pharmacy of Casablanca (FMPC), Hassan II University of Casablanca, Casablanca, Morocco; 4 Department of Microbiology, Immunology and Parasitology, College of Health Science, Addis Ababa University, Addis Ababa, Ethiopia; 5 Organisation Mondiale de la Santé, Bujumbura, Burundi; 6 National Public Health Institute, Ministry of Public Health, Bujumbura, Burundi; 7 Research Laboratory of Microbiology, Infectious Diseases, Allergology and Pathogen Surveillance (LARMIAS), Mohammed VI Faculty of Medicine, Mohammed VI University of Sciences and Health (UM6SS), Casablanca, Morocco; 8 Mohammed VI Higher Institute of Biosciences and Biotechnologies, Mohammed VI University of Sciences and Health (UM6SS), Casablanca, Morocco; 9 Mohammed VI Center for Research and Innovation, Rabat, Morocco; 10 Research Group for Genomic Epidemiology, National Food Institute, Technical University of Denmark, Kgs. Lyngby, Denmark; 11 Centre for Infectious Disease Control, National Institute for Public Health, and the Environment (RIVM), Bilthoven, The Netherlands; 12 Faculté de Médecine, Université du Burundi, Bujumbura, Burundi; Federal Medical Centre Abeokuta, NIGERIA

## Abstract

Despite remarkable progress towards measles elimination in Burundi, the country has seen a resurge in cases since 2019. Epidemiological investigations have been performed; however, it remained unclear if all measles cases involved in recent outbreaks were linked or caused by multiple independent events including introductions from other countries. Therefore, the objective of this study is to investigate the genetic diversity and evolution of measles virus (MeV) during the last large MeV outbreak in Burundi in 2024. The study was carried out on oropharyngeal swab samples collected from four neighboring health districts. Amplicon-based MeV sequencing was performed on the MinION Mk1D. Consensus sequences were generated from 18 isolates and phylogenetic and Bayesian evolution analysis including 152 closely related public genomes were performed. Results showed that all 18 newly generated whole-genome sequences belonged to the genotype B3. Phylogenetic analysis revealed a diverse population of MeV circulating in Burundi, with sequences divided into two separate clusters. The first cluster consisted of two sequences and was most closely related to Italian sequences, while the second cluster was more related to local transmission in the Great-Lakes region based on the N450 region. Based on whole-genome sequences, the remaining 16 whole-genome sequences from Burundi clustered with one sequence from the Netherlands. The most recent common ancestor of the sequences in the second cluster was estimated to be around the beginning of 2023 (between the end of 2022 and the end of 2023 using the 95% confidence intervals) by using Bayesian evolutionary analysis. Here, we provide the first batch of MeV whole-genome sequences generated on the African continent of the ongoing MeV outbreak in the Great Lakes region of Africa. Using these whole genome sequences, we demonstrated that measles viruses genotype B3 were already circulating in Burundi since the beginning of 2023 well before the outbreak in 2024 was noted. We also showed that the recurrent measles outbreaks in Burundi are ignited from different sources, showing that measles in Burundi is sustained by multiple introductions and emphasizing the importance of ongoing molecular surveillance for elimination efforts.

## Background

Measles is a highly contagious human disease caused by the measles virus (MeV), a single-stranded negative-sense RNA virus that belongs to the genus *Morbillivirus* of the *Paramyxoviridae* family [1,2]. Although MeV is antigenically monotypic, genetic variability in the hemagglutinin (H) and nucleoprotein (N) genes enables the classification of the virus into 24 genotypes (A, B1-B3, C1-C2, D1-D11, E, F, G1-G3, H1-H2). Genotype diversity has been used to monitor progress of measles elimination campaigns and since 2021, only genotypes B3 and D8 have been detected and reported to circulate worldwide [3]. Measles typically causes a red skin rash and is also associated with at least one complication in up to 20–30% of the cases a single complication can be observed [4–6]. The most serious complications include blindness, encephalitis, severe diarrhea, ear infections leading to otitis, and severe respiratory infections such as pneumonia [7].

Measles remains one of the most common causes of morbidity and mortality worldwide. In 2024 alone, it caused almost 11 million infections and 95,000 deaths globally [8,9]. The incidence of measles varies greatly globally, with the highest estimates of unconfirmed cases in geographic locations with low vaccine coverage and limited resources, particularly in Africa [10]. Here, measles remains endemic in many countries and according to the WHO, a total of 424,433 cases were reported in Africa in 2023 with large outbreaks occurring in countries such as the Democratic Republic of Congo (DRC), Nigeria, and Ethiopia [11].

Measles is a vaccine-preventable disease [12]. Measles virus vaccination was introduced in the Expanded Program of Immunization of Burundi in 1981 [13]. In line with the WHO regional goal towards measles elimination by 2020, the country had made commendable progress by maintaining a vaccine coverage of ≥90% for the first dose of measles vaccine between 2011 and 2015 [13]. Despite this progress made towards measles elimination in Burundi, the country has seen a resurgence of cases of the disease since 2019. The outbreak started in the Cishemere transit center of refugees from the Democratic Republic of Congo (DRC), located in the Cibitoke health district bordering the DRC and since it has spread to many other geographic locations within the country, particularly in health districts hosting refugee camps [14]. In 2024, 202 measles cases were reported, with 35.2% occurring in children younger than 9 months. Most cases were reported in northern districts, showing ongoing transmission in these areas and a higher risk among under-immunized children (National surveillance data, not published).

In recent years substantial progress has been made in strengthening in-country genomic surveillance capacity in Africa including Burundi. The National Reference Laboratory of Burundi has successfully generated and shared multiple whole-genome sequences of different pathogens, including monkeypox virus and SARS-CoV-2, demonstrating expanding national expertise and infrastructure for pathogen genomics within the country and have revealed how these viruses were introduced and subsequently spread within the country. However, molecular surveillance for measles virus is typically based on a short part of the genome, namely the 450 nucleotides at the end of the nucleoprotein coding region [3]. While this region is informative for genotyping, whole genome sequencing of MeV can provide a higher resolution to resolve transmission dynamics [15]. However, whole genome sequence data from the African continent remain limited. As of 28th November 2025, the Pathoplexus [16] database included 31 MeV genomes from Guinea, 8 from Uganda, 2 from Senegal, 2 from Sudan (dating from 1997), and 1 from Morocco. This limited representation underscores a significant gap in genomic surveillance, particularly given the substantial burden of measles outbreaks across the continent.

In Burundi, epidemiological investigations have been carried out to guide the development and implementation of measles outbreak control strategies [14,17,18]. These investigations showed that children aged 9–59 months were the most affected age group in detailed age-specific analyses, while overall 80% of cases occurred among children aged 9 years or younger. Most cases (77%) were either unvaccinated individuals or individuals who were unsure of their vaccination status [19]. Evidence-based strategies are crucial to control further introductions and widespread in-country and out-country transmission of the disease. The objective of this study was to investigate the genetic diversity and evolution of the MeV circulating in Burundi in 2024 to understand sources and modes of transmission for use as guidance for public health strategies.

## Materials and methods

### Study design, sample collection, and sampling sites

**Fig 1:**
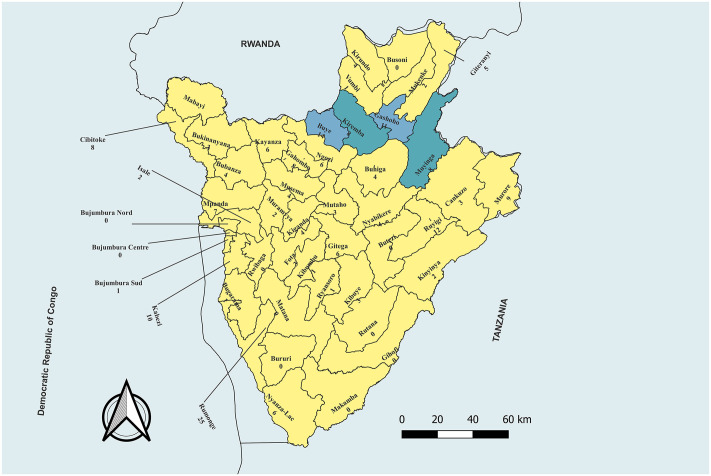
Mapping of the origin of the MeV sequences used in this study. Figs correspond to the number of laboratory-confirmed cases reported in each health district in 2024 (n = 202). The health districts concerned with this study are colored in red.

We conducted a cross-sectional study involving suspected MeV cases collected from health facilities in different health districts across the country between February and June 2024 (Table 1). In total, 18 oropharyngeal swab samples with sufficient volume and associated metadata were retrieved from the biobank of the National reference laboratory. We did not have access to all samples collected during this outbreak because sample collection and primary testing were done as part of routine national surveillance and not specifically stored for this study. They were collected from four different health districts with an active MeV outbreak at that time, namely Muyinga, Gashoho, Kiremba and Buye (Fig 1). The related data were accessed for research purposes between 1 January and March 2025. The authors did not have access to information that could identify individual participants during or after data collection. All data were anonymized prior to analysis.

**Table 1:** Epidemiological and genomic metadata of sequenced measles virus samples.

SEQUENCE_ID	Health district	Collection date	Age group (years)	Vaccination status	Genome coverage (%)	Genotype
1	Muyinga	Feb-24	1–4	Unvaccinated	99.73	**B3**
2	Kiremba	Feb-24	1–4	Unknown	95.42	**B3**
3	Kiremba	Feb-24	1–4	Unknown	90.59	**B3**
4	Kiremba	Feb-24	1–4	Unknown	97.60	**B3**
5	Kiremba	Feb-24	1–4	Unknown	97.56	**B3**
6	Muyinga	Feb-24	20–24	Vaccinated (1x)	99.38	**B3**
7	Buye	Jun-24	<1	Unvaccinated	93.09	**B3**
8	Buye	Jun-24	10–14	Vaccinated (2x)	97.56	**B3**
9	Buye	Jun-24	<1	Unvaccinated	98.17	**B3**
10	Buye	Jun-24	<1	Vaccinated (1x)	99.55	**B3**
11	Buye	Jun-24	<1	Vaccinated (1x)	99.47	**B3**
12	Buye	Jun-24	<1	Unvaccinated	99.73	**B3**
13	Gashoho	Jun-24	20–29	Unknown	98.10	**B3**
14	Gashoho	Jun-24	30–39	Unknown	95.41	**B3**
15	Gashoho	Jun-24	<1	Vaccinated (1x)	95.23	**B3**
16	Gashoho	Jun-24	30–39	Unknown	97.56	**B3**
17	Gashoho	Jun-24	<1	Vaccinated (1x)	95.41	**B3**
18	Gashoho	Jun-24	<1	Unvaccinated	97.33	**B3**

### Ethical approval

The ethical approval to conduct this study was given by the National Ethics Committee in Burundi (Reference number: CNE/10/2024). The national ethics committee provided a waiver for the consent from participants in this study. The samples were initially collected for the investigation and surveillance of measles.

### Nucleic acid extraction

Nucleic acids were extracted from 200µl of oropharyngeal swabs using the High Pure Viral Nucleic Acid Kit (Roche). Briefly, 200µl of the sample was mixed with 50µl of proteinase K and 200μl Binding Buffer followed by a heating step at 65^o^C for 5 minutes. After addition of another 100µl of Binding Buffer, the lysed sample was passed through a spin filter assembly, washed and eluted in 50µl of elution buffer. The extracted nucleic acids were stored at −20°C for further processing.

### PCR amplification, library preparation, and sequencing

After nucleic acid extraction, cDNA was synthesized in a two-step procedure involving denaturation and random priming using 0.5µl of random hexamers (50µM) (Promega), 0.75µl of 10mM dNTP mix, and 13.25µl of nucleic acids in the following conditions: 1 min 70°C, 4 min 65°C, 2 min 4°C. Then, cDNA synthesis was carried out using 5.5µl of master mix (4 µL of Superscript IV Buffer (5x), 1µl of DTT (100mM) and 0.5µl of Superscript IV Reverse Transcriptase) and 14µl of randomly primed RNA run as follows: 10 min 23°C, 30 min 50°C, 10 min 80°C.

The cDNA was subsequently amplified in four different amplification pools A, B, C and D. Briefly, 86µl of PCR Master Mix (20µl of Q5 reaction buffer 5x, 2µl of dNTP mix, 1µl of Q5 DNA-polymerase, and 63.5µl of PCR grade water) per sample was prepared and subsequently split in 4 different tubes. The pools A and B included 2µl of specific primers, 20.5µl of pre-PCR mix, and 2.5µl of cDNA while the pools C and D included 1 µL of specific primers, 22.75µl of pre-PCR mix, and 1.25µl of cDNA in a total volume of 25µl. PCR amplification occurred under the following settings: 98°C for 30 seconds, followed by 40 cycles of 98°C for 15 seconds, 64°C for 5 minutes.

After a normalization step involving pooling together 12.5µl of the PCR product from pool A and B and 3.5µl of the PCR-product from pool C and D for each sample, the pooled amplicons underwent a clean-up step using Ampure XP beads. We then quantified the pools using Qubit 4 Fluorometer (ThermoFisher) and took 100ng per sample for library preparation using the Native Barcoding Kit 24 v14 (Oxford Nanopore Technologies). Sequencing was performed on a MinION™ Mk1D (ONT) with R10.4.1 flowcells. Reads were basecalled with the high accuracy model on Dorado Basecall Server v7.4.13 (ONT).

### Data analysis and phylogenetic analysis

The bioinformatics analysis workflow began with quality control using fastp v1.0.1 [20] to trim low-quality sequences, while PCR primers were trimmed with Ampliclip [21]. The quality-controlled reads were mapped to the reference genome (NC_001498) using minimap2 v.2.28. Next, to generate a consensus sequence from the mapped measles data, Virconsens [22] was used with a minimum coverage cutoff of 30x. Mutation calling, quality checking, and genotyping were performed using Nextclade v3.9.1 [23]. The resulting consensus sequences were then aligned with all publicly available whole genome sequences and all publicly available N450 sequences, to have more African sequences included, using MAFFT v7.550 [24]. The resulting multiple sequence alignments were used as input to construct a maximum likelihood phylogenetic tree using IQ-TREE v2 [25] using the GTR + F + I + R4 and TIM + F + G4 models as best-fit models for the whole genome alignment and N450 alignment respectively, followed by visualization with Figtree and annotation with a custom R script.

### BEAST analysis

Based on the phylogeny of all publicly available whole genome sequences, a subset of close reference sequences was selected belonging to the Burundi specific clusters. These sequences were combined with the newly obtained sequences and aligned using MAFFT v.7.550 [24]. BEAST v.10.5.0 [26] was used to perform a time-resolved analysis on these 170 selected sequences using an uncorrelated relaxed clock and the HKY + G substitution model. The tree prior coalescent exponential growth was used and the MCMC was run for 200,000,000 iterations. Log files were analysed in Tracer v.1.7.1 and all ESS values were above the threshold (>200) and 10% burn-in was performed before the tree was visualised in FigTree v1.4.4 [27].

## Results

To better understand the genomic epidemiology as well as the genetic diversity and evolution of MeV in Burundi, we generated 18 whole genome sequences from 18 individual oropharyngeal swabs for which leftover samples had been stored. The samples were collected between the 2nd of February and the 27th of June 2024 and most of them were unvaccinated or the vaccination status was unknown. One individual received two vaccinations while five individuals got one vaccination of which four received the vaccination just before the date of rash onset while for one individual the date of vaccination was unknown (Table 1).

The sequences were generated from samples collected from 4 different health districts in the North of Burundi, including Buye (n = 6), Gashoho (n = 6), Kiremba (n = 4), and Muyinga (n = 2) (Fig 1). No sequences from Ruyigi and Murore could be generated since no samples from these health districts were available. The mean age of the sampled individuals was 7.6 years, ranging from 3 months to 33.1 years. Of the 18 individuals which were sequenced, 72.2% (n = 13) were from children under 5 years, and 27.8% (n = 5) of patients had already received the final dose of vaccination although in 4 of the 5 cases the final dose was administered 0–3 days before the rash onset. The average coverage of the generated sequences was 97.05% ranging from 90.59% to 99.73%. The analysis showed that all the sequences belonged to genotype B3.

**Fig 2:**
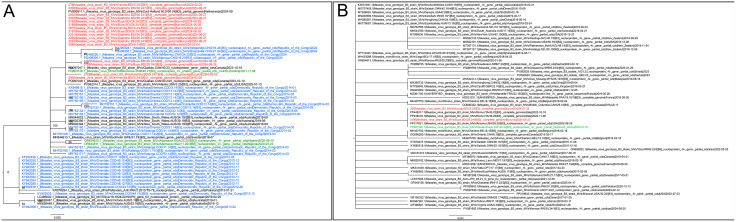
Zoom in of the two clusters in N450 region based maximum likelihood tree of newly generated MeV sequences and publicly available closely related N450 region sequences. (A) In red new sequences from Burundi are shown, in blue sequences from the DRC, in green sequences from other African countries and in black sequences from other countries. (B) In red new sequences from Burundi are shown, in Green a sequence from South Africa is shown and in black sequences from other countries. The scale bar represents the number of substitutions per site.

The results from the phylogenetic analysis based on the N450 gene of the newly generated MeV sequences from Burundi and publicly available N450 MeV genotype B3 sequences from other African countries showed that two different clusters of MeV are present in Burundi and that the Burundian sequences are clustering with sequences from other African countries such as the DRC, Zambia, and Nigeria (Fig 2).

**Fig 3:**
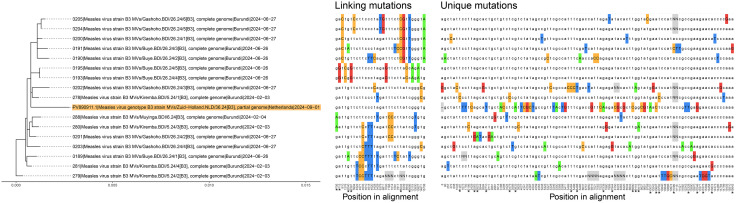
Zoom in of phylogenetic analysis of newly generated MeV whole genome sequences from Burundi and the sequence from the Netherlands (orange). All linking mutations and unique mutations based on the whole genome sequences as compared to the cluster consensus are plotted next to the phylogenetic tree and are highlighted by color and capital font.

However, while most N450 sequences from Burundi appeared to be identical, quite some mutations were observed when all WGS from Burundi were compared and we found considerable diversity within the big cluster with the sequences from Burundi. Each sub-cluster is associated with some linking mutations, but each sequence also has its own unique mutations (Fig 3).

**Fig 4:**
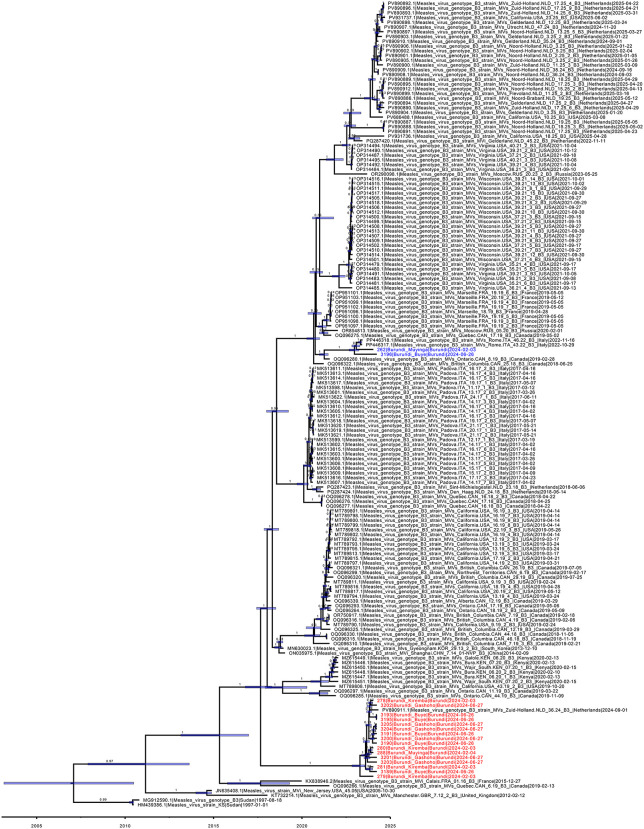
Maximum clade credibility tree for the sequences clustering with the sequences generated during this study. The MeV sequences from Burundi from the largest cluster are colored in red, the sequences from Burundi from the smaller cluster are colored in blue while the sequences from other countries are colored in black. The node bar indicates the 95% highest posterior density interval (HDP). The scale bar indicates the timing to the most common recent ancestor.

The phylogenetic inference based on whole genome sequences confirmed a diverse population of MeV circulating in Burundi, with sequences again divided into two different clusters. One of the two clusters contains two sequences from Burundi and are linked to European sequences from Italy, while the other contains 16 sequences from Burundi and one sequence from the Netherlands, collected in June 2024 (Fig 4).

The two samples which clustered with the Italian sequences were from two different health districts, namely Muyinga and Buye health district. The second cluster mainly consisted of sequences from Burundi, suggesting widespread occurrence of this viral cluster in Burundi. One sequence from the Netherlands clustered with the Burundian unique cluster. After consultation with the submitter of that sequence it appeared that the sample was collected in June 2024 from a patient who traveled to Malawi, four months after the data collection of some of the Burundi sequences within the cluster. Of note, the sequence from the Netherlands is divergent from the other sequences and does not have any shared mutation with the sequences from Burundi.

Based on the Bayesian evolutionary analysis, the time to the most common recent ancestor of the viruses mainly circulating in Burundi is in the beginning of 2023 (95% confidence interval between the end of 2022 and the end of 2023) (Fig 4) suggesting the outbreak has only started recently. However, the time to the closest related sequence to the sequences from Burundi and from the traveler from Malawi is estimated to be around 2018 making it unclear where and how the virus was circulating before it was detected in 2024 in Burundi (Fig 4).

## Discussion

This study aimed to investigate the current genomic epidemiology and evolution of MeV in Burundi through whole genome sequencing and phylogenetic analyses. Since 2019, the country has been experiencing recurrent measles outbreaks likely linked to intensified human movement, particularly refugees coming into Burundi from the DRC [19]. Ongoing conflicts, especially in the eastern part of the DRC, have also disrupted health services and hindered access to immunization programs. This led to large scale measles outbreaks in the DRC [28]. The largest one occurred from 2018 to 2020, with over 458,000 suspected cases and 8,000 deaths, primarily affecting children under five, with 41% of confirmed cases who received at least one dose of the measles vaccine [28]. This susceptibility to measles observed in this age group was also reported in other countries [19,29], and is likely the result of waning of passive immunity from maternal antibodies and incomplete vaccination of the children. Early waning of maternal antibodies observed at six months of age was reported in previous studies conducted elsewhere [29]. Previous reports have shown that some children with measles were vaccinated against measles, and this may be associated with seroconversion failure, either due to the suboptimal age at the first dose of vaccine, incomplete vaccination, or problems with the cold chain [30].

In Burundi, the routine measles vaccination schedule includes the first dose at 9 months of age and a second dose at 15 months, in line with WHO recommendations. In our dataset one individual with MeV infection was fully vaccinated while the other individuals were unvaccinated, the vaccination status was unknown or the vaccine was administered one to four days before rash onset. Typically, the MeV vaccine effectiveness increases during time after vaccination with an observed vaccine effectiveness of 96% one week after vaccination [31]. One case with a breakthrough infection was reported (5.5%) which is largely in line with numbers previously reported although the sample numbers in our cohort are too limited for solid conclusions [32–34].

To the best of our knowledge, this is the first time MeV whole genome sequences were generated on-site in Burundi. Genetic analysis of the 18 MeV sequences from Burundi showed that they all belonged to genotype B3. This MeV genotype was also reported in an outbreak that occurred in the Republic of Congo between 2023 and 2024 [35]. This is one of the two genotypes of MeV currently circulating globally, i.e., B3 and D8 [35]. According to the global epidemiological data published as of 2024, genotype B3 was predominantly reported in Africa, while the genotype D8 predominated in Europe [3,35].

Though the genomic sequences of measles variants from Burundi belonged to the same genotype, they formed two distinct clusters. Hence, at least two different introductions events with subsequent further spread seem to have occurred [36]. However, the presence of closely related sequences within the main cluster may also suggest local transmission following introduction events. Based on the time-resolved MCC tree it could be seen that the most recent common ancestor of the newly generated sequences dates back to the beginning of 2023. This suggests that the recurrent measles outbreaks in Burundi are likely ignited from different sources, showing that measles did not circulate for years already in Burundi but is most likely sustained by multiple different introductions. A substantial increase in measles cases linked to imported cases was also reported in other countries, such as Italy [37]. The observation of only two clusters of MeV circulating in Burundi could be due to the small sample size used in this study and the relative short time window in which the samples were collected. This study has some limitations, including the small number of available samples and the limited time distribution of samples, which may not fully represent the temporal diversity of circulating MeV.

Two measles sequences from Burundi clustered together with two sequences from Italy. This suggests that, at some point, MeV was transmitted between countries. The two sequences closely related to sequences from Italy were isolated from the Buye and Muyinga health districts. This link may be explained by the presence of a hospital, supported and run by Italians in Kiremba, Ngozi, with movement of people between Italy and that locality. Kiremba hospital serves people from many locations in Burundi; Buye and Muyinga health districts are in the catchment area of that hospital [38]. The transmission of MeV between medical staff and patients was reported in Italy in 2022 [39] and other countries [4], suggesting the possibility of such transmission events in health facility settings in Burundi. The two sequences from Burundi were obtained in February and June 2024. Their Italian counterparts were obtained earlier in late 2022. This period was followed by a surge of measles cases in Italy in 2023 [39], probably resulting from the expansion of initial cases and imported cases from other European countries without any link to the sequences from Burundi [40]. In a similar way to the two sequences from Rome, Italy, the small cluster of two sequences from Burundi did not expand. This might be the result of the limited sample size of this study or containment of the outbreak within the Kiremba hospital. Further investigations could find genetically related sequences, particularly in the area covered by Kiremba hospital.

Phylogenetic analysis based on the N450 gene including African sequences showed that the Burundi sequences are clustering with sequences from the Republic of the Congo, Zambia, Nigeria, and the DRC. This African link with the Burundi cluster based on N450 gene suggests that this large cluster of sequences from Burundi is likely most related to the African sequences. Analysis based on whole genome sequencing, however, showed only clustering with a sequence from the Netherlands with travel history to Malawi. Although there does not seem to be a direct link with this case, as the sequence from the Netherlands has many unique mutations not shared with the sequences from Burundi, this demonstrates that there is missing data from the African region, hampering more detailed source tracing and that additional sequence data from the African continent is essential to achieve a comprehensive overview.

National epidemiological data showed that a measles outbreak erupted in Cibitoke health district in Burundi in 2019 and spread to many other geographic locations within the country [19]. However, the most recent common ancestor of the sequences from MeV circulating in the four northern districts of Burundi in 2024 was estimated to be around 2023 by Bayesian evolutionary analysis using BEAST. The closest related sequences to the Burundian sequences, next to the Dutch patient with travel history to Malawi, is around 2018, showing a gap between 2018 and 2023. These data suggested that there were several introduction events which disappeared and were subsequently replaced by novel introductions. Unfortunately, older samples from before the 2024 MeV outbreak in Burundi have not been stored so no additional genomic information can be generated.

The obtained results demonstrated once again the benefits of whole genome sequencing in early detection and investigation of outbreaks with high resolution. It is crucial to scale up existing disease prevention, control, response, and surveillance strategies by leveraging and integrating whole genome sequencing technologies into the health system architecture, especially in Low and Middle-Income Countries (LMICs). The integration of genomic surveillance into the existing system has the potential to enhance surveillance through detailed tracking and tracing, guiding prevention and containment strategies of a threatening disease before widespread transmission. This would save lives and the economies of LMICs, enhancing the achievement of sustainable development.

## Conclusion

Through this study, we generated the first 18 publicly available whole genome sequences of MeV in Burundi. The phylogenetic analysis showed a diverse population of measles virus circulating in Burundi, all part of genotype B3, with sequences divided into two distinct clusters with at least two separate introductions in recent times. Providing higher resolution than classical epidemiologic approaches or partial genome sequencing, we demonstrated that the unique cluster was already circulating in Burundi before the outbreak started in 2024 but that it only dated back to the beginning of 2023, hence the same virus was most probably not circulating in the country since 2019 and several different introductions of MeV into Burundi occurred in recent years. Crucially, more whole genome sequencing from different time points is needed in Burundi and from neighbouring countries to better interpret the genomic epidemiology of measles virus in Burundi and the wider region.
